# Risk Factors for Coronary Drug-Eluting Stent Thrombosis: Influence of Procedural, Patient, Lesion, and Stent Related Factors and Dual Antiplatelet Therapy

**DOI:** 10.1155/2013/748736

**Published:** 2013-06-23

**Authors:** Krishnankutty Sudhir, James B. Hermiller, Joanne M. Ferguson, Charles A. Simonton

**Affiliations:** ^1^Abbott Vascular, Inc., Santa Clara, 3200 Lakeside Dr., Santa Clara, CA 95054-2807, USA; ^2^Center for Cardiovascular Technology, Stanford University, Palo Alto, CA 94305-5637, USA; ^3^The Care Group, LLC, St. Vincent Heart Center of Indiana, Indianapolis, IN 46290, USA

## Abstract

The complication of stent thrombosis (ST) emerged at a rate of 0.5% annually for first-generation drug-eluting stents (DES), often presenting as death or myocardial infarction. Procedural factors such as stent underexpansion and malapposition are risk factors for ST in patients. The type of lesion being treated and lesion morphology also influence healing after treatment with DES and can contribute to ST. Second-generation DES such as the XIENCE V everolimus-eluting stent differ from the first-generation stents with respect to antiproliferative agents, coating technologies, and stent frame. Improvements in stent structure have resulted in a more complete endothelialization, thereby decreasing the incidence of ST. Bioresorbable scaffolds show promise for restoring vasomotor function and minimizing rates of very late ST. Post-PCI treatment with aspirin and clopidogrel for a year is currently the standard of care for DES, but high-risk patients may benefit from more potent antiplatelet agents. The optimal duration of DAPT for DES is currently unclear and will be addressed in large-scale randomized clinical trials.

## 1. Introduction

Cardiovascular disease accounts for roughly one-third of human mortality throughout the world [1]. Although the last five decades have seen impressive improvements in the diagnosis, evaluation, and therapeutic strategies in patients with symptomatic cardiovascular disease the majority of deaths still occur in patients with occult disease. So-called “sudden cardiac death” (SCD) accounts for over half of all fatalities from cardiovascular disease, and remains a significant challenge to human health and longevity [2, 3]. This review will focus on a specific clinical and anatomic subset of coronary thrombosis that can lead to MI and SCD of thrombotic occlusion of a previously-stented coronary artery (stent thrombosis, ST) [4–6]. Although recognized and appreciated relatively recently [7–9], the study of the causes of ST has yielded invaluable pathophysiological observations and greatly accelerated the understanding of arterial clotting in general and its prevention. While a reliable and permanent solution for ST has remained elusive so far, considerable progress has been made in decreasing its incidence through optimal deployment techniques, improved stent design, and effective antiplatelet therapies.

## 2. Historical Perspectives

PCI dates back to the introduction of balloon angioplasty by Gruentzig [10]. Both early complications such as elastic recoil and acute vessel occlusion, as well as late restenosis from negative remodeling and to a lesser extent intimal hyperplasia, led to the development of stents made of bare metal (BMS), initially stainless steel, and later cobalt chromium alloys. Early in the BMS experience, subacute ST was a common occurrence until adequate stent sizing, high-pressure deployment, and postdilation were introduced [11], along with routine antithrombotic therapy [12]. However, restenosis, though less frequent with BMS compared to balloon angioplasty, was a persistent complication. In the early 2000s, drug eluting stents (DES) coated with polymers that released either sirolimus or paclitaxel emerged and decreased clinical and angiographic restenosis from 20–40% to <10% leading to their widespread use, soon accounting for >70% of all PCI procedures [13].

In contrast to balloon angioplasty, the phenomenon of “in-stent” restenosis is known to be caused by excessive neointimal proliferation at the site of the vessel injury, with little late vessel/stent recoil. DES reduce restenosis via inhibition of smooth muscle cell proliferation. However, since the metallic thrombogenic stent surface could potentially remain uncovered by endothelium for prolonged periods of time, the additional complication of ST reemerged. Although rates of ST during the initial year following implantation with first generation DES were similar to BMS, very late ST rates (beyond 1 year) were significantly greater. Very late stent thrombosis occurred at an approximate rate of 0.6% annually for first-generation DES, often presenting as a catastrophic event, with high rates of death (20–40%) or myocardial infarction (MI) (50–70%) [7, 14]. Further complicating the analysis of the real impact of this DES safety issue was their widespread “off-label” use, variability in the nature and duration of concomitant antiplatelet therapy, the long duration of followup required in clinical trials to quantitate risk of early, late, very late ST, and an extremely low frequency of events necessitating large sample sizes. In addition, the literature was confounded by a lack of universal definitions for ST, an issue addressed by the introduction of the Academic Research Consortium (ARC) criteria for definite and probable ST, which has standardized definitions enabling comparisons across trials and stent types [15].

## 3. Preclinical Correlates

Although human data are the best test of biocompatibility of DES, animal models can provide detailed systematic insights into tissue responses to DES under controlled conditions. Joner et al. have reported several pathological mechanisms that may contribute to ST, including factors observed in careful preclinical studies such as strut malapposition and hypersensitivity reactions [16]. Finn and colleagues compared the CYPHER sirolimus-eluting stent to a BMS control and showed a higher grade of inflammation in CYPHER stents at 180 days, with evidence of granulomas in 60% of arteries at 180 days [17]. These findings in CYPHER stents were confirmed in another study, where inflammation also was observed in TAXUS paclitaxel-eluting stents and BMS controls for the same platform but at a lower prevalence and with less intensity. Parastrut fibrin was more frequent in TAXUS than in CYPHER, and fibrin scores were greater in overlapped sections of both DES; fibrin was associated with stent malapposition [18]. In the rabbit iliac artery model, Finn et al. detected more luminal eosinophils but fewer para-strut giant cells with TAXUS stents at overlap compared to CYPHER stents at 1 and 3 months, with incomplete endothelialization [19]. 

The improvements in coating technologies, thinner struts, and more conformable designs using newer alloys may together be responsible for improved healing and enhanced endothelial coverage and consequently less ST in second-generation DES. In an animal model, four different DES were examined to determine the extent of neointimal stent coverage [20]. Rabbits received CYPHER, TAXUS, the ENDEAVOR zotarolimus-eluting stent, or the XIENCE V everolimus-eluting stent. Comparative analysis of endothelial coverage showed differences in arterial healing based on endothelial regrowth and recovery, favoring second-generation designs over first-generation stents. Coverage above struts remained poor in CYPHER, TAXUS, and ENDEAVOR (≤30%) relative to XIENCE V and Multilink (ML) VISION controls (≥70%) at 14 days, with no significant differences at 28 days. Significantly less endothelial strut coverage at 14 days was more apparent in earlier stent designs with CYPHER or TAXUS relative to the newer XIENCE V or ENDEAVOR. Furthermore, there were also differences in endothelial physiology, as reflected by reduced expression of PECAM-1 (an endothelial antigen PECAM-1 proven critical to endothelial homeostasis) in 14-day CYPHER, TAXUS, and ENDEAVOR, compared with XIENCE and ML VISION control stents, a finding that persisted at 28 days. As poor endothelial function and coverage may be risk factors for ST in humans [21], these preclinical findings may be relevant to differences in clinical outcomes across DES. 

## 4. Procedural Factors Predisposing to ST

Procedural factors such as stent underexpansion and malapposition are risk factors for ST [11]. Appropriate stent sizing and high-pressure (>14 atmospheres) stent deployment may minimize risk of ST. Although the value of intravascular ultrasound (IVUS) to ensure appropriate expansion has not been proven in randomized trials, it is often used to confirm stent apposition. A study using propensity-score matched analysis showed that patients undergoing IVUS-guided DES implantation had lower definite ST at 30 days and 12 months than those who had not received IVUS guidance [22]. In addition, stent length, multiple stents, incomplete lesion coverage leading to geographic miss, positive remodeling, persistent slow flow, residual stenosis and dissections have all been related to ST [23–27]. Late-acquired incomplete stent apposition, a phenomenon observed relatively more frequently with DES implantation compared with BMS implantation, may result from focal vascular remodeling and dissolution of thrombus. While there are conflicting reports regarding the possible impact of this IVUS finding on clinical outcomes, it is believed to be associated with late adverse cardiac events including late ST [28]. 

## 5. Lesion and Patient-Related Factors

The type of lesion being treated and lesion morphology also influence healing after treatment with DES and can contribute to ST development. Lesion characteristics such as necrotic cores associated with acute coronary syndromes may delay healing, since strut coverage may not be uniform, leading to toxicity from the drug or polymer [29]. In addition, stenting of bifurcation lesions or instent restenosis lesions [7, 30, 31] and revascularization of more complex lesions appears to increase the risk of ST. Several patient-related factors have been associated with the development of ST with DES, including primary stenting in acute MI or an acute coronary syndrome, smoking, diabetes mellitus, renal failure, and low ejection fraction [7, 30–34]. A recent pooled analysis showed that the risk of ST was inversely related to age, with younger patients showing a higher incidence [35], a noteworthy finding given that older patients are known to have a higher incidence of bleeding after PCI [36]. 

With first-generation stents, concerns were raised regarding increase in ST related to use in complex patients (so-called “off-label” use). Data from the EVENT registry [37], an analysis from the National Heart, Lung, and Blood Institute Dynamic Registry [38] and another single centre study of BMS and DES patients [39] showed that onlabel stent procedures were associated with lower risk of MI, death, and ST (particularly late and very late events) compared to offlabel stent procedures. Despite these concerns about late stent thrombosis with DES, long-term followup of randomized DES versus BMS trials showed that after accounting for ST events after procedures for restenosis, which occur more commonly after BMS than DES, the overall incidence of ST is not increased with DES compared to BMS [40], and the overall late rates of death and MI are similar with DES and BMS [41]. Of note, the benefits of DES in reducing restenosis and subsequent adverse events appear to offset a small excess risk of very late ST even with first-generation DES [42].

## 6. Stent Factors

Late ST, more likely to occur with DES than with BMS, may result from the gradual release of the antiproliferative agent, effectively inhibiting endothelialization of the stent struts, allowing them to remain a nidus for platelet aggregation and thrombus formation. Angioscopic assessment in patients 3–6 months after stent deployment showed that BMS were completely endothelialized, whereas most DES were not, and thrombi were frequently visible in DES [43]. Postmortem studies conducted 40 months after the placement of DES confirmed poor endothelialization in 45% of cases [16]. First-generation DES use may thus be associated with a small increase in ST and in high-risk patient and lesion subsets, an increased risk of death or MI compared with the use of BMS [44]. ST after BMS typically occurs within the first-month following implantation, though rarely later. Data derived from studies of first generation stents have demonstrated that ST after DES can be delayed, with an annual incidence of 0.2-0.3% in patients with noncomplex coronary artery disease [45] and 0.4–0.6% in real-world patients [46]. 

Second-generation drug-eluting stents are designed to improved stent delivery, efficacy, and safety. The second-generation XIENCE EES has been shown to be superior to a first-generation TAXUS PES in preventing the clinical manifestations of stent thrombosis in label patients [47], real-world patients [48], and those with acute coronary syndrome [49]. Second-generation stents differ from first-generation stents with respect to the antiproliferative agent, the coating technologies employed toward the polymer layer (which acts as a reservoir for controlled drug delivery), and the stent frame [50]. Improvements in stent structure may result in better stent apposition to the vessel wall, improved endothelialization, and reduced platelet aggregation and thrombus formation, thereby reducing the incidence of ST [51]. The extent of endothelial coverage is dependent on object thickness [52], so thin strut designs may have lower risk for developing ST, as endothelial cell coverage would occur more rapidly compared to thicker struts. Endothelial coverage is the greatest in stents with the least strut/polymer thickness, namely, XIENCE V (89 *μ*m) and ENDEAVOR (96 *μ*m) compared with TAXUS LIBERTE (113 *μ*m) and CYPHER (153 *μ*m), where endothelial coverage was uniformly poor [20]. An optimal combination of thin fracture-resistant cobalt-chromium struts, low dose of everolimus elution [53], and well-known thromboresistant noninflammatory proprieties of the fluorinated polymer [54, 55] may contribute to the lower rates of both early ST and late ST with XIENCE V. While low rates of late ST and very late ST have been reported with other new DES such as the ENDEAVOR RESOLUTE Zotarolimus-eluting stent [56, 57], and XIENCE is the only second-generation DES to consistently show low early ST rates, even in “all comer” populations, when compared to other first- or second-generation DES [56, 58]. Of note, in a network meta-analysis of 49 trials including 50,844 patients, cobalt chromium EES showed the lowest rate of ST among DES within 2 years of implantation, with the surprising finding of reduced stent thrombosis compared with bare-metal stents [59]. Further, in another meta-analysis of eleven randomized controlled trials of 16,775 patients, the XIENCE V EES compared with a pooled group of paclitaxel-eluting stents, sirolimus-eluting stents, and zotarolimus-eluting stents is associated with a significant reduction of definite ST, an effect that appears early and increases in magnitude through at least 2 years [60]. A similar finding emerged in a recent mixed-treatment comparison analysis of 117,762 patient-years of followup from randomized trials, where among DES types, there were considerable differences, such that EES, SES, and ZES-R that were the most efficacious, while the XIENCE V EES was the safest stent [61]. Of interest, in patients with diabetes, a further analysis analysis of 22,844 patient years of follow-up from randomised trials, relative differences were observed among the drug-eluting stents, such that the XIENCE V everolimus eluting stent was the most efficacious and safe [62]. In a recent analysis from real-world patients with acute myocardial infarction (AMI) in the XIENCE V USA trial, AMI patients treated with XIENCE V had low rates of ST, TLR, and TLF at 1 year, similar to non-AMI patients [63]. These findings were further confirmed in a recent network analysis of 12,453 patients with ST-segment elevation myocardial infarction, where the XIENCE cobalt-chromium EES was associated with lower cardiac death, MI, and ST rates than BMS [64].

In the LEADERS trial, where the BIOMATRIX flex biolimus-eluting stent with a biodegradable polymer was compared to the CYPHER select sirolimus-eluting stent, ST rates were not significantly lower with the BIOMATRIX flex stent at either 9 months [65] or 3 years [66]. Early data with the NOBORI biolimus-eluting stent, another metallic stent with a biodegradable polymer, showed absence of ST at 9 months [67], but in the randomized, controlled noninferiority trial COMPARE II, a rate of 0.8% at 1 year was noted for the biolimus-eluting stent, not different from that observed for the everolimus-eluting stent (1.0, *P* = 0.58) [68]. Of note, a case report of late ST, 5 months after NOBORI implantation, associated with accelerated neoatherosclerosis and early manifestation of neointimal rupture, suggests that larger studies and continued vigilance are necessary [69]. These findings suggest that biodegradable polymers on metallic platforms offer no incremental benefit over durable polymer DES. The wholly bioresorbable scaffold ABSORB everolimus-eluting stent, which resorbs completely over an approximately 2 year period, has shown promise in early studies, restoring vasomotor responsiveness as early as 1 year after implant, with zero ST to 5 years in the first-in-human Cohort A trial of ~30 patients [70], zero ST at 2 years in the initial 44 patients in the Cohort B trial [71], and low ST rates (<1%) to 1 year in the first 450 patients in the absorb extend trial, which allowed overlap, included more unstable angina patients, and expanded into emerging geographies in Asia-Pacific and Latin American countries [72]. Inherent in the promise of this novel technology is the ultimate disappearance of the scaffold, which should in theory eliminate the long-term risk of device-related thrombosis.

## 7. Pharmacological Therapy for Platelet Inhibition

Current recommendations for dual antiplatelet therapy (DAPT) in PCI patients include aspirin and a thienopyridine, based on randomized trials showing reduced ST rates with aspirin plus clopidogrel or ticlopidine, compared to aspirin alone or aspirin plus warfarin [73–75]. Post-PCI treatment with aspirin and clopidogrel is currently the standard of care for most patients undergoing PCI, except higher risk patients, such as those with acute coronary syndromes, who might benefit from more potent antiplatelet agents such as prasugrel and ticagrelor, though at the cost of increased bleeding [76, 77]. Clopidogrel is a prodrug that is converted via the CYP2C19 enzyme into its active metabolite. Polymorphisms in the gene encoding the CYP2C19 allele, as well as heightened onclopidogrel platelet reactivity, have been associated with adverse clinical events in patients undergoing PCI [78, 79]. The US Food and Drug Administration recently amended the label for clopidogrel, warning about reduced effectiveness in patients who are poor metabolizers [80]. However, at the present time, the clinical value of genetic testing for CYP2C19 polymorphisms or platelet reactivity is uncertain. Of note, the Gravitas trial showed no benefit for doubling the standard daily dose of clopidogrel from 75 to 150 mg per day after PCI in patients with heightened on-treatment platelet reactivity [81].

## 8. Duration and Discontinuation or Interruption of DAPT

The ideal duration of antiplatelet therapy is unclear. For BMS-treated patients, while a minimum of 2–4 weeks of dual-antiplatelet therapy is recommended, 12 months may be optimal, especially in patients with acute coronary syndromes. Premature thienopyridine cessation after DES placement is strongly associated with ST, with the highest risk within the first 30 days [82]. It is generally recognized that elective dental, endoscopic and other procedures within the first year after DES implantation should be performed without discontinuation of either aspirin or clopidogrel, and elective surgery should be deferred if possible. However, in an analysis of the initial 5054 patients in XIENCE V USA, a condition-of-approval, postmarket, single-arm, real-world trial of the XIENCE V DES, Krucoff et al. have shown that DAPT interruption appears to be safe beyond 1 month in standard risk (on label) patients and beyond 6 months in an unselected (all-comers) population, suggesting that this second-generation stent may indeed have an enhanced safety profile [83]. In the most comprehensive analysis of DAPT interruption to date, Stone et al. recently studied 11,219 patients from the XIENCE V family of trials and showed that ST within 2 years is infrequent following PCI with XIENCE V (0.75%); ~50% of events occurred within 30 days; most ST episodes (80.0%) within 2 years occurred in patients on DAPT; and ~70% of ST episodes occurred without prior DAPT discontinuation. Importantly, the ST rate in patients discontinuing DAPT at any point was similar to that in patients who never discontinued DAPT [84].

Current recommendations are for 12 months of dual antiplatelet therapy in most patients after PCI [85]. Arguments for prolonged dual antiplatelet therapy remain unsupported by clinical evidence; Park et al. have shown that dual antiplatelet therapy for a period longer than 12 months in patients who had received DES was not significantly more effective than aspirin monotherapy for cardiac death/MI or ST [86]. Two years of dual antiplatelet therapy after coronary stenting were no more effective than 6 months of treatment at reducing ischemic events but doubled the rate of major bleeding in the PRODIGY trial (PROlonging Dual-antiplatelet treatment after Grading stent-induced intimal hyperplasia study), in which 2013 patients who underwent stenting were studied [87]. A considerably larger prospective, multicenter, randomized, double-blind trial is currently ongoing to assess the effectiveness and safety of 12 versus 30 months of DAPT in subjects undergoing PCI with DES and BMS [88]. Of interest, a recent study by Naidu et al. [89] was the first to suggest that DAPT interruption after 30 days does not increase the incidence of ST within the first postprocedure year when using the XIENCE V EES. Further, a more recent analysis of pooled data from XIENCE V postapproval trials from Mehran et al. [90] demonstrated that ST rates following DAPT interruption were very low (0.19%) in patients who interrupted DAPT beyond one month and not different from those who had never interrupted DAPT (0.26%). Although these studies do not advocate for discontinuation of DAPT before 1 year (or, if necessary, after 30 days), they help confirm recent trials such as the PRODIGY study and serve as the basis for further studies of extremely short-duration DAPT.

## 9. Conclusions

ST represents a major complication of DES implants, usually leading to either cardiac death or MI. Preclinical studies have shown that inflammation, parastrut fibrin, and endothelial coverage vary between stents, and more biocompatible polymers in newer DES may have improved endothelial coverage and thus less ST. The risk of ST in an individual patient is related to numerous factors that include patient and lesion complexity, suboptimal stent deployment, adherence to and duration of dual antiplatelet therapy, and stent type and design (see Table 1). There is emerging evidence that second-generation stents, particularly XIENCE V, have significantly lower ST rates compared to first generation stents. Various components of the newer DES, including thinner struts, more conformable metallic alloys and designs, and improvements in coating technologies, as well as optimal drug dosage and pharmacokinetic elution profiles, may all contribute synergistically to the preclinical and clinical evidence of enhanced safety. Stent designs of the future hold promise, and there is considerable clinical interest in the wholly bioresorbable scaffold, ABSORB for both restoration of vascular function and potentially minimal very late ST. Treatment with DAPT for a year is currently the standard of care for DES, but more potent antiplatelet agents such as prasugrel and ticagrelor may be beneficial in high-risk patients. DAPT interruption appears safe beyond 30 days in standard risk patients and beyond 6 months in an all-comers population that received the XIENCE V DES. The optimal duration of DAPT for DES is unknown; recent data indicate that short-term therapy may well be sufficient for real-world patients treated with XIENCE, a finding that should be systematically confirmed in large-scale randomized controlled trials.

## Figures and Tables

**Table 1:** Risk factors for stent thrombosis.

Procedural	Lesion related	Patient related	Stent related	DAPT related
Stent underexpansion	Necrotic cores	Acute MI	Antiproliferative agent	Premature discontinuation
Stent malapposition	Bifurcation lesions	Acute coronary syndrome	Coating technologies	Interruption
Stent length	Instent restenosis	Diabetes mellitus	Polymer biocompatibility	CYP2C19 polymorphisms
Multiple stents	Chronic total occlusion	Renal failure	Strut/polymer thickness	Platelet reactivity
Geographic miss	Diffuse disease	Low ejection fraction	Stent structure	Antiplatelet drug type
Positive remodeling	Small vessel disease	Younger age	Drug dosage	Duration of therapy
Persistent slow flow		Smoking		
Residual stenosis				
Dissections				
